# Interfacing aptamers, nanoparticles and graphene in a hierarchical structure for highly selective detection of biomolecules in OECT devices

**DOI:** 10.1038/s41598-021-88546-4

**Published:** 2021-04-30

**Authors:** Carlotta Peruzzi, Silvia Battistoni, Daniela Montesarchio, Matteo Cocuzza, Simone Luigi Marasso, Alessio Verna, Laura Pasquardini, Roberto Verucchi, Lucrezia Aversa, Victor Erokhin, Pasquale D’Angelo, Salvatore Iannotta

**Affiliations:** 1 IMEM - CNR Institute of Materials for Electronics and Magnetism, Parco Area delle Scienze 37/A, 43124 Parma, Italy; 2 Physics Department and Ph.D. School on Material Science and Technology, University of Parma, Parma, Italy; 3 Department of Chemical Sciences, University of Naples “Federico II”, 80126 Napoli, Italy; 4 Chilab - Materials and Microsystems Laboratory, DISAT, Politecnico di Torino, Chivasso, Turin, Italy; 5 Department of Industrial Engineering, University of Trento, 38123 Trento, Italy; 6 IMEM - CNR Institute of Materials for Electronics and Magnetism, Trento Unit, c/o Fondazione Bruno Kessler, Via alla Cascata 56/C, Povo, 38123 Trento, Italy; 7 Present Address: Indivenire s.r.l., 38123 Trento, Italy

**Keywords:** Biophysics, Biotechnology, Biomarkers, Materials science, Nanoscience and technology

## Abstract

In several biomedical applications, the detection of biomarkers demands high sensitivity, selectivity and easy-to-use devices. Organic electrochemical transistors (OECTs) represent a promising class of devices combining a minimal invasiveness and good signal transduction. However, OECTs lack of intrinsic selectivity that should be implemented by specific approaches to make them well suitable for biomedical applications. Here, we report on a biosensor in which selectivity and a high sensitivity are achieved by interfacing, in an OECT architecture, a novel gate electrode based on aptamers, Au nanoparticles and graphene hierarchically organized to optimize the final response. The fabricated biosensor performs state of the art limit of detection monitoring biomolecules, such as thrombin-with a limit of detection in the picomolar range (≤ 5 pM) and a very good selectivity even in presence of supraphysiological concentrations of Bovine Serum Albumin (BSA-1mM). These accomplishments are the final result of the gate hierarchic structure that reduces sterich indrance that could contrast the recognition events and minimizes false positive, because of the low affinity of graphene towards the physiological environment. Since our approach can be easily applied to a large variety of different biomarkers, we envisage a relevant potential for a large series of different biomedical applications.

## Introduction

Medical science is currently taking advantage from a wide set of organic electronic devices implementing, for instance, medical diagnostics/care^1,2^ as well as in neuromorphic applications^3^. This is because bioelectronic devices based on biocompatible organic conductors are endowed with a mixed ionic-electronic conduction that makes them suited for the biointerfacing and allows the design of electronic devices able to implement an efficient ion-to-electron transduction, useful for their interfacing with the standard electronics. The latter property is pivotal for a special class of transistors, i.e. the Organic Electrochemical Transistors (OECTs), which are ideal candidates for several applications in bioelectronics^2^. OECTs’ operation relies on a change of the device output current upon the interaction of their active channel with charged species dispersed in an electrolyte, in direct contact with it. Such interaction is mediated by the action of a gate electrode immersed in the same electrolyte. OECTs are currently experiencing a renewed interest in bioelectronics. Indeed, we are witnessing a development of novel strategies, straightforward and powerful at the same time, conferring the needed selectivity for the recognition of ionic species dispersed, at low concentrations, in complex biological fluids^4^ . This is because OECTs lack of intrinsic selectivity: the benchmark organic transducer they are based on, i.e. the poly(3,4-ethylenedioxythiophene):polystyrene sulfonate (PEDOT:PSS), accomplishes an efficient transduction of all charged species in the electrolytic medium due to a de-doping effect occurring upon their gate voltage-driven diffusion into its bulk^5^. Hence, several strategies towards selectivity have been proposed in literature and particularly developed in the last years. OECT specificity has been achieved basically by pursuing two approaches, the first one consisting of device interfaces functionalization^6,7^, while the second one involves novel measurement protocols^8,9^. The first strategy, representing the most popular approach in literature, has led to a large variety of devices upon functionalization of the gate electrode or of the device channel^10^. The development of OECT-based immunosensors, implemented via gate functionalization, for label-free transduction of bio-recognition events is really promising for promoting a combined improvement of selectivity/sensitivity^11,12^. The second approach, indeed, even though not very common, seems to be appropriate for discriminating different analytes in biological fluids. It is based, for example, on the use of AC measurements where dopamine detection can be implemented by applying a gate voltage pulse and collecting the OECT current/transconductance responses, which show phase angle shifts that are dependent on the analyte concentration in the electrolyte^9^, or on the appropriate choice of transconductance, a parameter expressing the amplification capability by transistors, as figure of merit for the selective detection of different analytes mixed in a physiological environment. In this case, the transconductance as a function of the gate voltage shows multiple peaks whose positions have been associated to each specific analyte, specifically dopamine, ascorbic acid and uric acid, dissolved in an electrolytic medium^8^. The demanding requirement for even better performing OECTs biosensors in terms of selectivity and Limit of Detection (LoD) can be addressed by further implementing novel and promising bioreceptors. There is an increasing interest in aptamers, massively used in impedimetric aptasensors^13^. Aptamers are synthetic nucleic acid sequences (short single-stranded DNA or RNA) endowed with specific bio-recognition abilities through their tight binding with non-nucleic acid targets. Their strong affinity with specific target molecules is promoted by different secondary structures, such as stems, loops or tertiary motifs as G-quadruplexes, that favor binding with target analytes through different physico-chemical interactions, including van der Waals interactions, hydrogen bonding and $\pi -\pi$ stacking of aromatic moieties^14,15^. In this way, aptasensors may accomplish the detection of analytes up to the femtomolar^16^ and attomolar level^17^. The aptameric approach in the context of OECT biosensing is up to now limited to few recent papers. Chen et al. have explored the detection of glycan expression, i.e. an integral membrane protein ruling cell-to-cell interaction, on living cancer cells down to 10 cells/μL^18^. They have exploited a complex gate electrode interface and, analyzing the OECT time response upon subsequent injection of analyte aliquots in the gate electrolytic medium, observed an effective gate voltage shift upon recognition events. Saraf et al., based on the same type of data analysis of an effective gate voltage shift^19^, were able to detect epinephrine down to the picomolar level by exploiting epinephrine binding aptamers immobilized on Ti/Au gate electrodes^20^. Liang et al. have demonstrated the possibility of selectively detect adenosine triphosphate (ATP) with ultrahigh sensitivity down to the concentration of 10 pM^21^. The real time approach is expected to be time effective and, consequently, able to enhance the sensor swiftness. However, it realizes the well-known competition between swiftness and sensitivity in sensors, the latter being the minimum variation of analyte concentrations able to produce a variation of the sensing parameter. In fact, this protocol provides a short incubation time, implying a less effective coordination and recognition of the analyzed biomolecule. In addition, the memory of the past binding events during the subsequent additions of analyte aliquots in the electrolyte is expected to reduce the electrode surface still available for biorecognition events, thus reducing the sensor sensitivity. Here we report a detailed study aimed at a very sensitive and selective detection of thrombin, a protein involved in the blood coagulation, down to the picomolar level. Our experiments have been implemented on a OECT aptasensor based on PEDOT:PSS channels and specific gold nanoparticles/polyethylene multi-layers graphene (AuNPs-PMLG) gate electrodes^22^ functionalized by immobilizing the Thrombin Binding Aptamer (also named TBA or TBA15). This aptamer, carrying the sequence 5′GGTTGGTGTGGTTGG3′, adopts a stable chair-like, antiparallel G-quadruplex (G4) structure and strongly inhibits fibrin clot formation by tightly binding to thrombin^23^. It has attracted huge attention as effective thrombin activity modulator, representing one of the most extensively studied systems in the development of thrombin-targeting assays^24^, and it has been also incorporated in nanosystems and nanoparticles^25–27^. The performance of our sensing devices has been carefully investigated by means of several techniques aimed at testing the fabrication process quality for the AuNPs-PMLG/TBA gate electrode. We discuss the effectiveness of such electrode in determining a hierarchic geometry, which is expected to synergistically combine a large surface area for the direct immobilization of aptameric layer with a graphene-based multilayer. The latter is characterized by the highest mechanical strength, an excellent electrical conductivity, an high surface area, a good biocompatibility and, above all, a low affinity towards undesired molecules that can aspecifically adsorb on metallic electrodes, allowing to reduce false positive results and to reach an even marked selectivity upon detecting very low levels of thrombin in presence of supraphysiological amounts of Bovine Serum Albumin (BSA), i.e. at a concentration of 1mM, being BSA the most abundant serum protein with a physiologic concentration of about 600 μM^28,29^. Our approach, taking advantage of an AuNPs-PMLG composite, is also reinforced by the choice of a novel sensing parameter, the $\Delta ratio$, a parameter expressing the relative change of drain-source current for Thrombin concentration, that is expected to give a direct correlation between the binding events and the channel current change^30^. The use of the $\Delta ratio$ parameter allows excluding spurious effects due to single components of the fabricated aptasensor, such as aptamers, graphene and AuNPs^31^.

## Results

### Structure of the transistor

**Figure 1:**
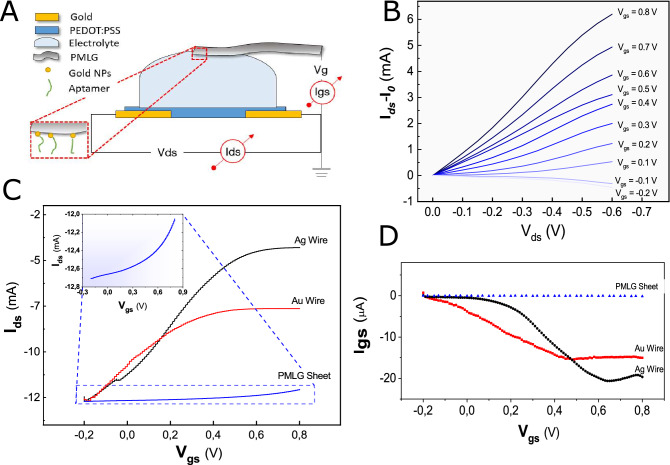
(**A**) layout of the PMLG gated OECT; (**B**) related $I_{ds}$—$I_0$ versus $V_{ds}$ (from 0 to − 0.6 V) recorded using PBS 10 mM as electrolyte (here, $I_0$ is the current measured at $V_{ds} = 0$ V) and fixing $V_{gs}$ in the range − 0.2 to 0.8 V, step 0.1 V, for each curve; (**c**) PMLG OECT’s transfer characteristics obtained at $V_{ds}=-0.25$ V for different types of gate electrodes and (**D**) related gate currents (Ag wire -black line, Au wire—red line and PMLG sheet—blue line). The inset of (**C**) shows a magnification of PMLG response.

We fabricated an OECT sensor integrating functionalized polyethylene multi-layer graphene (PMLG) gate electrodes in a standard organic electrochemical transistor structure (Fig. 1A). Consisting of a conductive multi-layers graphene (MLG) deposited on a flexible and insulating substrate made of a low density polyethylene (LDPE) film, the gate electrode well combines the flexibility and the electrical properties of graphene, necessary in biological applications. The PMLG thin film consists of an uncontrolled and not ordered over-layer of graphene sheets with different dimensions, probably due to the deposition procedure, with a structure intermediate between graphene and graphite (Supplementary Materials—Fig. S1). This morphological observation has been further supported by Raman spectroscopy (Supplementary Materials—Fig. S2).

### Preliminary OECT characterization

The transistor response when gated with the bare PMLG electrode has been characterized using PBS (a phosphate buffered saline solution, pH 7.4, mimicking the extracellular media) as electrolyte.

The output characteristics of the PMLG OECT (Fig. 1B) acquired monitoring the channel current ($I_{ds}$) as a function of the source-to-drain voltage ($V_{ds}$) and of the gate-to-source voltage ($V_{gs}$), shows trends typical for a device operating in the depletion mode^32^, at low voltage operating conditions, which is an important requirement for electronic device applications in the biological field. Excluding the very low voltage ($V_{ds}$ < 0.1 V), there is a clear discrimination between curves corresponding to different $V_{gs}$. This means that even small $V_{gs}$ changes affect the device output current ($I_{ds}$), showing that PMLG is a suitable material for gating an OECT. However, in sensing applications it is necessary to achieve a highly stable and fast responding system, in which the temporal response ideally depends only on the ions transport through the electrolyte. To achieve this, the transit time of holes in the PEDOT:PSS channel must be low enough to be considered negligible. We estimated this parameter to be 52.20 μs for our system indicating that holes transport in the channel is not the limiting factor in the OECT temporal response^33^ (Supplementary Materials—Fig. S3).

Corresponding transfer curves, obtained monitoring the evolution of $I_{ds}$ at fixed $V_{ds}$ while varying $V_{gs}$ ( Fig. 1C) indicate that the PMLG gate electrode (blue curve and inset) gives rise to an $I_{ds}$ modulation, although much smaller with respect to that of gold (Au) and especially silver (Ag) wire electrodes, hence it is a suitable material for sensing applications. This small amplification presumably depends on the electrode reactivity towards analytes dispersed into the electrolyte, as indicated by the gate current behavior (Fig. 1D), which represents an efficient way to control and study the faradaic and non-faradaic contributions of the electrodes^34^. Indeed, depending on the specific gate potential window used to collect the OECT response, the electrode can manifest a sustained faradaic behavior (Ag vs. dissolved salts), corresponding to a reduced potential drop at the electrode/electrolyte interface, but also a more (Au vs. dissolved salts) or less (PMLG vs. dissolved salts) effective polarizable character towards the gate electrolyte, causing a significant potential (and gate current) drop at the gate electrode/electrolyte interface. From our characterization it is worth to note that the strong contribution of $I_{gs}$ in the case of Ag and Au electrodes (reflecting in a sustained $I_{ds}$ response), may potentially hide any other events of feeble entity occurring in the proximity of the gate electrode, including the interaction between a target molecule and the bioreceptor deposited on the gate. Conversely, PMLG, which is mostly inert against saline buffers in the gate voltage window used for our experiments, may enhance the ability to detect any other event (eventually coming from non-metallic bioanalytes) in the gate electrode proximity, making it better suited for the pursued sensing application.

### PMLG-gold nanoparticles-aptamers

The decoration with metallic nanoparticles (NPs) is an effective method to enhance the conductivity and the sensing performance of electrodes. In addition, NPs are often used as anchoring elements for different compounds, being able to efficiently link chemical groups by generating strong bonds. However, it is extremely important to have NPs with a homogenous dimensional distribution to ensure an efficient decoration and controllable properties of the final electrode. We adopted the Frens protocol for the realization of AuNPs with finely tunable size, shape and electrical properties^35^. AuNPs, prepared using this seed-induced growth method (described in "Synthesis and characterization of gold nanoparticles" section) have a narrow dispersion (between 10 and 20 nm) and an average diameter of about 13 nm (Supplementary Materials—Figs. S4 and S5). These NPs have been then deposited on the surface of the PMLG electrode substrate as described in the “Materials and methods” section (AuNPs electrophoretic deposition section). The resulting decorated PMLG electrode has an estimated percentage of surface coverage of 15$\%$ which has been further confirmed by the narrow intense peak in the Energy Dispersive X-ray analysis (EDX) (Supplementary Materials—Fig. S6).

**Figure 2:**
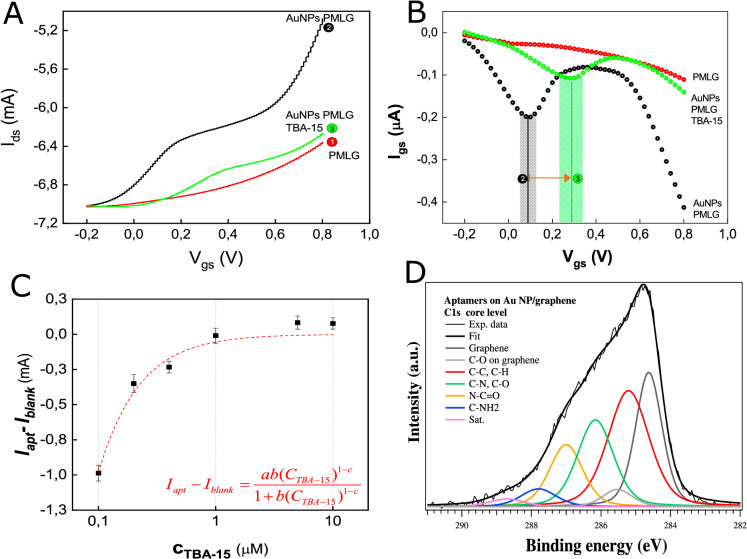
(**A**) comparison between the transfer curves for the different gate electrodes (i.e. unfolded TBA15-functionalized AuNPs-PMLG gate electrode (concentration 1 μM—green lines), bare PMLG (red lines) and AuNPs-PMLG (black lines)) and (**B**) related gate currents. Relative peaks are indicated with (2) for the AuNPs-PMLG and (3) for TBA15-functionalized AuNPs-PMLG gate electrode; (**C**) Optimization of the $I_{apt}-I_{blank}$ parameter as a function of the aptamer concentration (fitting curve:Langmuir isotherm,red line); (**D**) C1s core level XPS analysis of PMLG/AuNPs/TBA-15. Single components are described in the legend.

The decoration with AuNPs enhances the OECT amplification capability, showing a higher current modulation (black curve in Fig. 2A)) with a profile quite different from the nearly-linear trend of the bare PMLG gate electrode (red line). This enhancement, already reported by Sensi et al. for carbon based gate electrodes decorated by AuNPs^36^, reveals an electron transfer process between AuNPs and the graphene electrode^37^ and is demonstrated by the appearance of a well-defined peak at 0.1 V in the gate current profile (Fig. 2B, black curve), as well as by a step-like trend of Ids between 0.2 and 0.6 V (Fig. 2A, black curve). As already mentioned, NPs are widely used as anchoring elements for the functionalization of electrodes. Our intent was the immobilization on gold of large densities of the thrombin binding aptamer TBA15 as bioreceptor for detecting low Thr concentrations. The decoration of the quasi-2D PMLG electrode with nanostructures provides an enhancement in the aptamer loading and offers a large surface area for the self-assembled monolayer. In addition, the out-of-plane structure provided by AuNPs decoration is expected to minimize the steric hindrance generated by TBA15 conformational changes taking place during the Thr recognition^38^. A robust and “semi-covalent” bond linking the aptamer onto the electrode surface is desirable in order to ensure reliable and stable electrical measurements. For the immobilization of TBA on the electrode (see "Aptamer preparation and functionalization" section), we took advantage of the very high affinity between thiol groups (inserted at the 3′-end of the aptamer) and AuNPs. In turn, the 5′-end of the aptamer was modified with a ferrocene residue (Supplementary Materials—Fig. S7), introduced as a redox probe, sensitive to the aptamer conformational changes occurring upon variation in the electron transfer processes^39^, similarly to what shown in the case of an electrochemical biosensor used for $K^+$ detection^40^. Our design was based on the well-known ability of thrombin to induce structuring and stabilize the G-quadruplex conformation of TBA15 and its analogues^41,42^. To confirm the effective binding of the aptamers onto the AuNPs, X-ray photoelectron spectroscopy (XPS) studies were performed on three samples: the bare PMLG electrode, the AuNPs-PMLG electrode and the AuNPs-PMLG decorated with the TBA15 (Fig. 2D).

Only carbon and oxygen species are present in the bare PMLG sample, while gold signal has been found for the other two AuNPs modified gate electrodes (Supplementary Materials—Figs. S8–S11 and in Table 1 and 2). In the functionalized electrode we observed the presence of nitrogen, phosphorous and sulphur, i.e. elements present only in the aptamer. The S2p core level is located at 161.80 eV (Fig. S11A), suggesting the formation of a chemical bond with AuNPs^43,44^. C1s peak (Fig. 2D) shows a complex line-shapes, with features from the PMLG substrate, located at 284.62 and 285.52 eV^45^, while components related to the aptamer can be clearly identified such as C–C/C–H species, (at 285.21 eV)^46^, C–N in the nucleobases and C-O in the sugars of the oligonucleotide backbone (at 286.1 eV)^47^. Moreover, further features are present at 287.00 eV, related to N–C=O bond in the two basis^48^ and at 287.79 eV that can be attributed to the C–NH$_2$ groups in the guanines^49^. An additional peak at 288.71 eV is likely arising from a shakeup electron promotion process, typical of large organic molecules^50^. A more detailed analysis of XPS spectra (reported in Supplementary Materials, Figs. S8–S11) confirms the presence of TBA15 on the AuNPs-PMLG, with AuNPs functionalized by the thiol group.

The transfer and the related gate current curves for this functionalized electrode, acquired prior to carry out the Thr recognition, are reported in Fig. 2A,B (green curves), respectively. In the $I_{gs}$ curve, we observe that the peak attributed to the electron transfer process between gold and graphene (peak (2) at 0.1 V) before the aptamer functionalization is then replaced by a broad and less intense peak centered at 0.3 V (peak (3)). The broadness and the shift to higher voltage values are presumably the result of the change in the electron-transfer kinetics mediated by AuNPs, due to the self-assembly of negatively charged aptamers on the electrode surface^51,52^ in combination with the formation of a competitive electron transfer process between ferrocene (Fc) and graphene^53,54^. The aptamer immobilization on the decorated electrode impedes the electron exchange between AuNPs and the electrode^52^, promoting the transfer process borne by the Fc label. This fact represents an indirect evidence of the aptameric functionalization. However, the low peak intensity suggests that the Fc label transfer cannot compensate the intrinsic screening effect due to the aptameric functionalization, whose major effect is to markedly increase the electron transfer resistance of the electrode^52,55,56^. This fact leads to a lower $I_{ds}$ modulation that becomes comparable with that of the bare PMLG gate electrode. This screening effect arises from the variation of the capacitive EDL due to the gate functionalization^11^.

The weaker $I_{ds}$ modulation with respect to the AuNP-PMLG case is a further clear evidence of the aptamer functionalization (in the previous case, responsible of the enhancement in the transfer curve). However, in the case of the aptamer functionalization, we observed that the $I_{ds}$ modulation depends on the concentration used in the incubation process of the initial oligonucleotide solution, suggesting a close connection between the aptamer coverage on the gate and the final amplification of the OECT. To determine the optimal surface concentration of TBA15, i.e. the one maximizing the device sensitivity, the procedure based on six different aptamer concentrations (CTBA-15) has been followed (Aptamer preparation and functionalization section). Figure 2C shows the evolution of the difference between the $I_{ds}$ current (at $V_{gs}$ = 0.8 V) measured at a given aptamer concentration ($I_{apt}$) and the $I_{ds}$ response for a bare PMLG gate electrode ($I_{blank}$). A complete saturation is expected if nearly all the AuNPs are decorated by TBA15, saturating the entire gate electrode surface. This implies that the only contribution to the OECT response is due to the electron transfer induced by the aptamer. The difference between $I_{apt}$ and $I_{blank}$ decreased with the increase of the aptamers’ concentration, demonstrating that the more the AuNPs were covered with the oligonucleotides, the higher was the shielding effect of the nanoparticles on the $I_{ds}$ modulation. The formation of a mono-layer of non-interacting aptamers was confirmed by fitting all acquired data with a Langmuir isotherm fit (LangmuirEXT1 function, OriginPro 2018 software), whereas the Langmuir adsorption model is generally suitable for describing the chemisorption process and has been largely used to describe the surface coverage by a monolayer of small-molecule adsorbates upon their interaction with an adsorbent surface^57^. This also indicated that the sought optimal concentration was 1 μM.

### Thrombin detection

**Figure 3:**
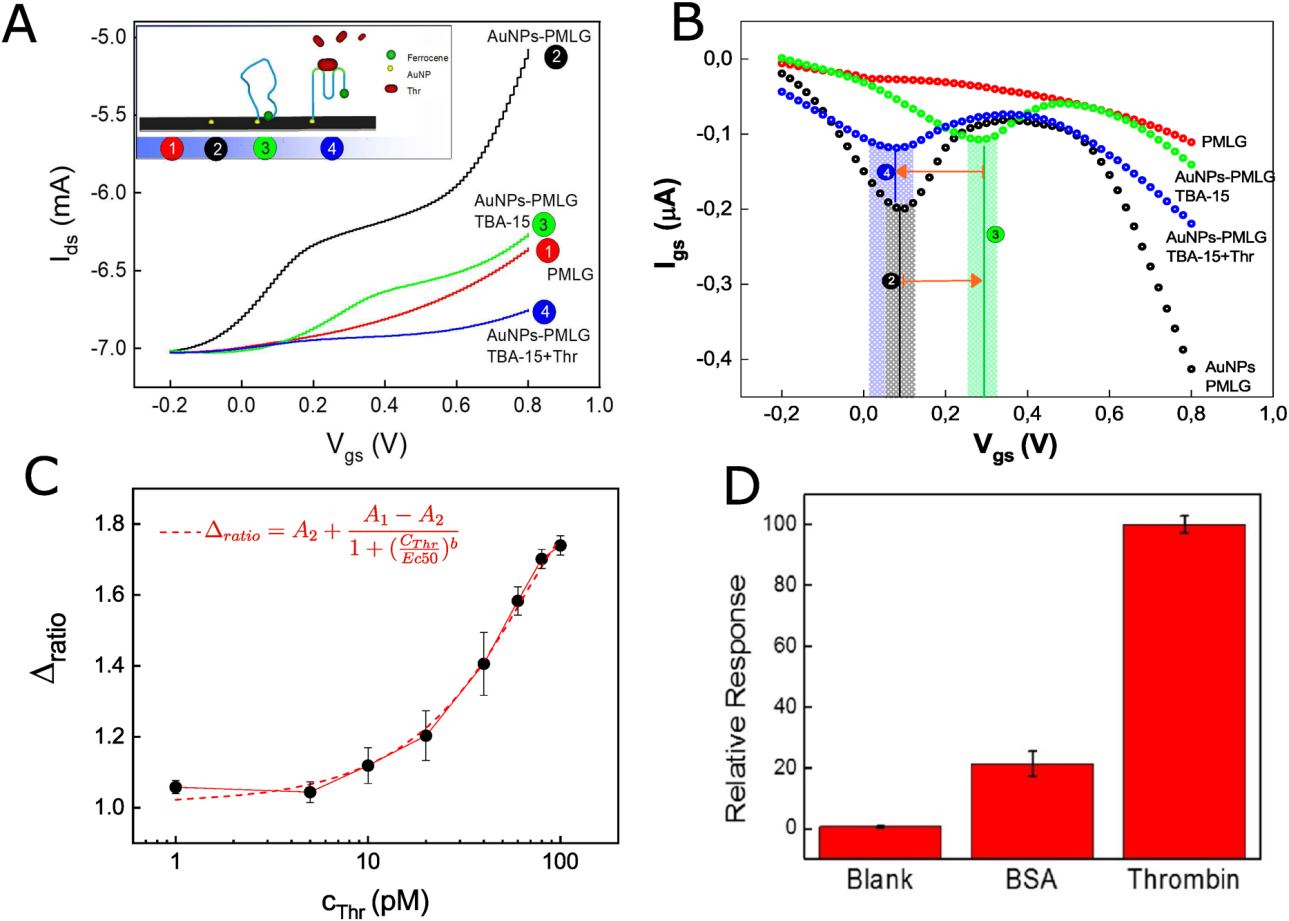
(**A**) comparison between typical transfer curves obtained for thrombin-incubated and non-incubated gate electrodes (TBA15-functionalized AuNPs-PMLG, blue line; unfolded TBA15-functionalized AuNPs-PMLG, concentration 1 μM -green line; bare PMLG, red line, and AuNPs-PMLG, black line. In the inset: proposed detection method) and (**B**) related gate currents. Relative peaks are indicated with (2) for the AuNPs-PMLG, (3) for TBA15-functionalized AuNPs-PMLG and (4) for TBA15-functonalized AuNPs-PMLG gate electrode; (**C**) typical device response to different concentrations of thrombin ($C_{Thr}$) described by introducing the $\Delta ratio$ parameter; (**D**) selectivity test: the signal recorded in the presence of higher concentration of BSA (1 mM) is compared with the one obtained in presence of very low thrombin concentration (100 pM).

A control transfer curve has been recorded for the optimal aptamer concentration in order to monitor the transition between folded and unfolded TBA15-functionalized AuNPs-PMLG gate electrode (Fig. 3A, blue line). The acquired curve has been compared with the one reported in Fig. 2A (green curve), whose features have been previously discussed in "PMLG-gold nanoparticles-aptamers" section. Similarly to the case of the AuNPs-PMLG electrode (reported as black line in Fig. 3B), the corresponding gate current shows a peak centered at 0.1 V that is characterized by a much lower intensity (peak 4, blue line in Fig 3B). The conformational change of the aptamer upon thrombin recognition, consisting of a transformation from a random coil, flexible structure to a rigid one, i.e. the compact G-quadruplex, promotes the distancing of the ferrocene 5’-ending group from the gate electrode surface (inset of Fig. 3A). As a result, the inhibition of the electron transfer observed at 0.3 V (green curve in Fig. 3B), tales place^53,54,58^. The resulting $I_{ds}$ modulation, due to the steric hindrance caused by the presence of thrombin, is weaker than that obtained in the case of bare PMLG gate electrode (blue and red lines in Fig. 3A, respectively). Steric hindrance, in fact, reduces the surface portion of gate electrode available for an effective interaction with the electrolyte, thus increasing the electron transfer resistance^52^. This marked variation in the transfer curves in terms of both shape and modulation can be considered as the direct effect of the thrombin detection. To evaluate the linear range and the detection limit of the OECT based biosensor against thrombin, the calibration curve upon testing different thrombin concentrations (from 1 to 100 pM), was determined. The device response, reported in Fig. 3C, is expressed as the $\Delta ratio$ as function of the thrombin concentration ($C_{Thr}$). From its definition, $\Delta ratio$, which is equal to 1 if no detectable binding event takes place, is more precise than the current modulation parameter used elsewhere^11^, since it excludes from the analysis all possible effects deriving from the aptamer, AuNPs and gate substrate and it only allows the evaluation of the thrombin coordination to the OECT sensor response. The $\Delta ratio$ versus $C_{Thr}$ curve can be fitted by a sigmoidal function (red curve in Fig. 3C) where A1 and A2 are the minimum and the maximum analytical response, b is the slope of the inflection point and EC50 is the concentration corresponding to $50\%$ of the maximum signal. EC50 extracted from the fitting procedure is equal to 48.91 pM (correlation R2 of 0.99154). The limit of detection (LoD) of the device was calculated as $\Delta blank$ + 3$\sigma$ (IUPAC standard), where $\Delta blank$ is the average of the OECT response for blank signal (i.e. the sensor response in absence of the analyte), $\sigma$ is the standard deviation related to the blank measurements and 3 is a numerical factor realizing a confidence level of 99.86$\%$. Accordingly, the calculated LoD is 5.000±0.087 pM. It is worth noting that a concentration of 5pM corresponds to a $\Delta ratio$ very close to1 (Fig. 3C). This value falls within the range for the early detection of coagulative events potentially associated to tumor growth, even though is not the best value reported in literature for thrombin detection. Selectivity tests, aimed at further proving the efficacy of the proposed aptasensor ( whose final aspect is reported in Supplementary Materials Fig S12), were performed in presence of bovine serum albumin (BSA). Albumin is the most abundant plasma protein, normally present at a concentration of about 600 μM^28,29^. The aptasensor selectivity has been evaluated in worst conditions, i.e. at a supraphysiological BSA concentration of 1 mM. The $\Delta ratio$ assessed in the presence of BSA was normalized with respect to the $\Delta ratio$ corresponding to 100 pM of thrombin and reported in percentage, together with a blank measurement performed after the interaction of 100 pM thrombin with a non-functionalized AuNPs-PMLG gate electrode (Fig. 3D). The upmost aptasensor specificity against thrombin is realized, showing the device a relative response of 100$\%$, even if some non-specific BSA recognition, characterized by a relative response of about 20$\%$, can be observed too. On the other hand, the blank measurement shows a very small signal related to non-specific interactions between thrombin and the unmodified AuNPs-PMLG surface (relative response of about 0.4$\%$). This, in turn, demonstrates once again that the obtained signals are due to the selective interaction between TBA15 and thrombin. It is worth noting that our system presents a high ratio between non-specific and specific interactions, even under severe testing conditions. A.H. Loo et al. reported a graphene based impedimetric aptasensing of thrombin whose selectivity against BSA was tested using a ratio between BSA and Thr concentration of 5/1^30^. Their results demonstrated a variation in the response of about 1/2 (Rct(Thr) $=$ 0.7 versus Rct(BSA) $=$ 1.1). A rather similar variation in specific and non-specific results has been obtained using a pure gold electrode in which this ratio is about 1/3^59^. The introduction of AuNPs with their large specific surface area on a metallic disk electrode^60^provides better selectivity, obtaining a ratio of 1/8 between BSA and Thr signals, but in this case the ratio between tested concentrations is $10^6 /1$. Indeed, we tested the optimized version of our sensor using a more severe concentration condition $10^7 /1$(1 mM of BSA vs. 100 pM Thr) and, nevertheless, we were able to obtain a ratio between relative responses of 1/5.

## Discussion

The optimized hierarchical organization achieved by interfacing, on graphene sheets deposited on a Polyethylene flexible film, Au nanoparticles and aptamers has been the key to develop OECTs with unprecedented selectivity as demonstrated by studies on Thrombin used as a test biomolecule. The aptasensor performance took full advantage of the properties of the developed materials and interfaces where the innovation is the gate electrode that combines a multilayer graphene sheet, almost inert towards physiological fluids, with electrocatalytically active AuNPs. The large surface area of nanoparticles has been chosen also for easily immobilizing a larger number of aptamer molecules on the surface of the electrode leaving them the ability to change conformation, making them a useful alternative for signal amplification, while the underlaying graphene sheets, being practically inert, give the advantage of minimizing false positive results. These relevant device innovations relying on the materials and interfaces here implemented were critical for the successful monitoring of thrombin, a benchmark biomolecule used as a significant case study, up to the picomolar level, moreover showing a noteworthy selectivity and absence of nonspecific interactions even when exposed to supraphysiological concentrations of BSA, an interfering protein present in blood plasma. We believe that these results further qualify OECTs as very promising devices for biomedical applications in a perspective of biocompatible, cheap and easy-to-handle systems that could contribute to the development of point of-care diagnostics. We envisage that our approach could be profitably expanded to a large number of different biomarkers, being basically limited only by the aptamers design and fabrication.

## Materials and methods

### OECTs fabrication and characterization

A standard 2” (100) p-doped Si wafer finished with 1 μm of $SiO_2$ is used as substrate for the fabrication of an array made of eight nominally equivalent OECTs. A Ti (20 nm)-Au (100 nm) bilayer is deposited using an e-beam evaporator (ULVAC EBX-14D) for the source and drain electrodes, following the procedure reported in Ref^22,61^. More in detail, for the metal patterning process, AZ1518 photoresist (PR) is used in combination with a two-steps wet etch process for the selective removal of the metals. Two distinct solutions are used for the etching: $KI: I_2: H_{2}O = 4 g: 1 g: 40 ml$- for Au layer and $HF: H_{2}O_{2}: H_{2}O = 1: 1: 20$—for the Ti layer. Successively, for the PEDOT lift-off process, AZ 5214E PR is used in the photolithographic step. An $O_2$ plasma activation of the surface is performed prior to deposit the conductive polymer layer. A PEDOT:PSS solution (Clevios PH1000, ethylene glycol 20:1 in volume, Sigma Aldrich, and DBSA 0.05$\%$, Sigma Aldrich) is double spin-coated on the wafer, to obtain a 260 nm-thick film. After annealing at 150 °C in vacuum for 90 min, the wafers are left in dimethylsulphoxide (DMSO) at 60 °C for 1 h for the lift-off step, then are rinsed with isopropyl alcohol (IPA). A Sylgard 184 poly(dimethylsiloxane) (PDMS, Sigma Aldrich) well was applied to the wafer by stick and stamp technique employing a molding process on a polymethylmethacrylate (PMMA) master.

The graphene gate electrode consists of conductive multi-layers graphene (MLG) deposited on a flexible and insulating substrate made of a low density polyethylene (LDPE) film. Briefly, the polyethylene multi-layer graphene (PMLG) films were produced through exfoliation of nanographite through thermal and ultrasonic treatments ensuring mechanical lamination of the material. The obtained powder has been suspended in an alcoholic solution and deposited on LDPE surface by means of drop casting^62^. The as-prepared gate electrode has been characterized using high-resolution scanning electron microscopy (ZEISS field emission gun scanning electron microscope) and Raman spectroscopy (Renishaw InVia Qontor) (Supplementary Material—Figs. S1 and S2).

The OECT’s electrical characterization has been carried out using two source measure units (NI PXle-4138/9) controlled by a customized ad hoc LABVIEW code and analyzed by means of OriginLab software. Our electrical set up ensures reliable and standardized data acquisition. The typical reproducibility of OECTs is shown in Supplementary Material-Fig. S13. To ensure a proper contact between the electrolyte and the gate the PMLG electrode was set horizontally, parallel to the polymeric channel and directly in contact with the electrolyte solution, fixing the distance between the gate electrode and the OECT channel at 1mm using micromanipulators in order to keep the system stable and reproducible. The volume of electrolyte (PBS 10 mM) during all characterizations was set to 200 μL. OECTs output curves consist of channel current ($I_{ds}$) recording as a function of the source-to-drain voltage ($V_{ds}$) ranging between 0 and − 0.6 V, scan voltage step and rate of 0.05 V and 5 s, respectively. During negative $V_{ds}$ scans, the gate-to-source voltage ($V_{gs}$) was kept constant. Different values of $V_{gs}$ were investigated, fixed between 0 and 1 V with steps of 0.1 V. Corresponding transfer curves were obtained recording *I*_*ds*_ at a fixed value of $V_{ds}$ (− 0.25 V) while varying $V_{gs}$ between − 0.2 V and 0.8 V, steps of 0.01 V and scan rate of 5 s. This methodology is common for all the reported transfer curves, including those obtained using bare, gold nanoparticles (AuNPs), AuNPs-TBA and AuNPs-TBA-Thr graphene gate electrodes, for the total of 22 measured gate electrodes.

### Synthesis and characterization of gold nanoparticles

AuNPs were synthesized following the Frens protocol^35^. Briefly, a solution of tetrachloroauric acid $HAuCl_4$ (50 mL of 10–2$\%$ p/p, solution I) and a solution of sodium citrate (10 ml of 1$\%$ p/p, solution II) were prepared. The first one was heated to its boiling point ($\approx$ 100 °C) under magnetic stirring. After that, 1 mL of solution II was added to the former solution. The tetrachloroauric acid reduction by sodium citrate is complete after five minutes of reaction at the boiling temperature. Before any characterization, the AuNPs suspension has been centrifuged to remove impurities and nanoparticles aggregates (Rotina 380, Hettic—12000 rpm for 20 min at room temperature). After removing the supernatant, AuNPs dispersion has been stored in the dark at room temperature. To characterize freshly synthesized AuNPs, UV-vis spectroscopy and transmission electron microscopy (TEM) have been carried out using a standard Thermo-Fisher system and JEOL JEM 2200 EX, at an accelerating voltage of 200 kV. The histogram of the particles size distribution and their average diameter were obtained by measuring a batch of 500 AuNPs using the ImageJ Fiji Software. The samples were prepared by evaporating a drop of gold colloids onto dedicated carbon-coated copper grids and allowing them to dry in the air. The hydrodynamic diameter distribution of the AuNPs in solution was determined by Dynamic Light Scattering (DLS). The DLS measurements were carried out with a 90 Plus Particle Size Analyzer from Brookhaven Instruments (Holtsville, NY) with a 1.2 mW HeNe laser as light source, a scattering geometry at 90° in 2$\Theta$ and s polarization. The detector was a single mode fiber coupled avalanche Geiger module (SensL) with a time resolution of 60 ns. The single photon signal was correlated with a 480 ns resolution correlator. Each sample was allowed to equilibrate for 10 min prior to starting the measure and five independent measurements, each one during 60 s, were performed and averaged. A dilution with Milli-Q water allowed obtaining a final AuNPs concentration close to 100 μg/mL. The Zeta potential was determined using a Zeta Plus from Brookhaven Instruments (Holtsville, NY) operating at applied voltage of 4 V and 4 runs of ten cycles per samples were performed to ensure measurement repeatability. Each reported value was the average of the four runs.

### AuNPs electrophoretic deposition

The electrophoretic deposition (EPD) method was exploited to deposit AuNPs on the PMLG gate electrode surface with two source measure units (NI PXle-4138/9) controlled by a customized LABVIEW code. Before the EPD, the PMLG gate electrode was sonicated in isopropanol for 10 min in order to clean it. The PMLG electrode was immersed into the AuNPs suspension and was used as anode, together with a chrome electrode (5 mm × 10 mm) employed as cathode. The two electrodes were placed at a distance of about 1 cm and a voltage of 40 V was applied at the anode for 15 min. Finally, the PMLG gate electrode decorated with AuNPs (AuNPs-PMLG) was thrice rinsed with Milli-Q water (> 18 M$\Omega$ cm) and dried in air.

### AuNPs-PMLG gate electrode characterizations

To investigate the quality of the AuNPs thin film deposited on the PMLG electrode surface, high-resolution scanning electron microscopy (ZEISS field emission gun scanning electron microscope) was performed (electron beam acceleration voltage of 5 keV at working distance of 3.9 mm). The AuNPs-PMLG gate electrode was also characterized by Energy Dispersive X-ray spectroscopy (EDX) performed with an Oxford-Cambridge 360 Stereo Scan working at 200 KeV, in order to study the graphene nanocomposite surface coverage. The PMLG surface was investigated by scanning the electron beam over the area of interest and simultaneously acquiring the EDX spectrum, with the aim of reconstructing the spatial distribution of the C and Au signals.

### Optimization of the aptamers concentration

The 15-mer thrombin binding aptamer, used as bioreceptor with a sequence 5’ Fc-G-G-T-T-G-G-T-G-T-G-G-T-T-G-G- C6-SH 3’, was synthesized by BIOMERS (Germany) and characterized by HPLC and MALDI-TOF analyses (data given by the provider). The C6-SH at the 3’ end is a thiol group separated from the oligonucleotides sequence by a linear linker containing six carbons, while Fc at the 5’ end stands for a ferrocene residue covalently linked to the aptamer sequence and used as redox label.

### Aptamer preparation and functionalization

For the TBA immobilization onto the AuNPs-PMLG gate electrode, 10 μl of solutions with different aptamer concentrations (0.1, 0.2, 0.4, 1, 5 and 10 μM) in sterile water were heated at 90 °C for 5 min to allow the aptamer destructuring, increaseing the thiol group accessibility. The samples were successively dipped in a bath of cold water for 15 min to freeze the aptamer conformations. Each solution was drop casted on the surface of an AuNPs-PMLG gate and dried in ambient conditions for 4 h. Finally, the prepared TBA-functionalized AuNPs-PMLG electrodes were washed twice with sterile water to remove the unbound aptamers. After the aptamer immobilization, each gate electrode was dipped in a solution of 6-mercapto-1-hexanol (MCH, purchased from Merck, Germany), with a concentration of 100 μM, for 15 min at room temperature under gentle stirring to minimize possible unspecific binding (post-blocking agent). Two washing steps with sterilized water were finally carried out.

### TBA-functionalized AuNPs-PMLG gate electrode characterization

X-ray photoelectron spectroscopy (XPS) has been performed to confirm formation of the gold-thiol binding between aptamers and AuNPs on the PMLG surface. Photoelectron analyses were performed in a UHV chamber equipped with a non-monochromatized Mg K$\alpha$ X-ray source (photon energy 1253.6 eV), at normal acceptance. A VSW HA 100 electron analyzer (with PSP electronics) was used to analyze the energy of the emitted photoelectrons (resolution of 0.8 eV). All the core level binding energies (BE) were normalized to the Au 4f7/2 core level signal (at 84.0 eV), obtained from a sputtered gold surface. The core level analysis has been performed by Voigt line-shape deconvolution after the background subtraction of a Shirley function. The typical precision for each component’s energy position is ±0.05 eV. The maximum uncertainty for the full width at half-maximum (FWHM) is less than $\pm 2.5\%$, while for the area evaluation it is about $\pm 2\%$.

### Thrombin detection

To evaluate the sensing performance of the OECT-based aptasensor against thrombin (Thr) protein, a calibration curve was made at different concentrations of Thr. The purified human $\alpha$-thrombin from its lyophilized form (Sigma-Aldrich) was dissolved in its buffer (50 mM Tris, 1 mM EDTA, 1 mM $MgCl_2$, 150 mM KCl, pH 7.4), obtaining a stock solution with a concentration of 1 μM. Sample solutions with different Thr concentrations were prepared from this stock solution, obtaining the following concentrations: 1, 5, 10, 20, 40, 60, 80 and 100 pM. The last step of the analytical procedure consisted in the recognition of thrombin by the immobilized aptamers on the AuNPs-PMLG gate electrode. To do this, 10 μl of each Thr solution at the desired concentration was drop casted on the gate electrode surface and incubated overnight at 4 °C. The electrode was then washed twice with sterile water to remove unspecific protein adsorption.The device response is expressed using the parameter $\Delta ratio$:

1 $$\begin{aligned} \Delta _{ratio} = \frac{I_{ds,(TBA-15-Thr)} - I_{ds,(AuNPs-PMLG)}}{I_{ds,(TBA-15)} - I_{ds,(AuNPs-PMLG) }} \end{aligned}$$

where $I_{ds}$ is measured at $V_{gs}$ = 0.8 V, $I_{ds,(TBA-15-Thr)}$ is the $I_{ds}$ measured after the incubation of thrombin, $I_{ds,(TBA-15)}$ is the *I*_*ds*_ measured after aptamer immobilization and $I_{ds,(AuNPs-PMLG)}$ is the channel current measured with the AuNPs-PMLG electrode in absence of TBA15 (and represents the blank measurement), at each Thr concentration, and reporting it as a function of all the analyzed concentrations. The sensing response was tested at least three times to evaluate the mean and the standard deviation values for every combination of thrombin doses.

### Evaluation of the aptasensor selectivity

The evaluation of the OECT-based aptasensor against thrombin was tested by drop casting 10 μl of a solution of bovine serum albumin (BSA, Biowest, USA) with a concentration of 1 mM in PBS buffer (137 mM NaCl, 2.7 mM KCl, 10 mM $Na_2HPO_4$, 1.8 mM $KH_2PO_4$, pH 7.4) on the surface of a TBA-functionalized AuNPs-PMLG gate electrode. The choice of the buffer was dictated by the analysed protein and followed the manufacturers recommendations. The choice of a Tris buffer for $\alpha$-thrombin allowed maintaining the conformational integrity of this very sensitive serine protease, here analyzed at very low concentrations, avoiding thus even a partial denaturation of the aptamer. The protein was incubated overnight at 4 °C following the same procedure employed for thrombin and, finally, the electrode was washed twice with sterile water to remove proteins in excess. It is worth noting that the two buffers have the same pH and very similar overall ionic strength, both reproducing physiological buffers. The formation of aspecific bonds between thrombin and the gate electrode surface was investigated performing a “blank” measurement. To this aim, 10 μl of a 100 pM thrombin solution were drop casted on the AuNPs-PMLG gate electrode surface without aptamers modification and the thrombin was incubated overnight at 4 °C. The electrode was washed twice with non-pyrogenic, distilled water to remove the Thr excess and the OECT response was based on transfer characteristics recorded using the same parameters employed for the calibration curve.

## Supplementary information

Supplementary material 1 (docx 4559 KB)
