# Supplementary material for: Interfacing aptamers, nanoparticles and graphene in a hierarchical structure for highly selective detection of biomolecules in OECT devices
Source: Sci Rep. 2021 Apr 30;11:9380. doi: 10.1038/s41598-021-88546-4 (PMC8087810; doi:10.1038/s41598-021-88546-4)
Supplement: Supplementary file 1 — Supplementary material 1 (docx 4559 KB) [file 41598_2021_88546_MOESM1_ESM.docx]

Interfacing Aptamers, Nanoparticles and Graphene in a hierarchical structure for highly selective detection of biomolecules in OECT devices.

Carlotta Peruzzi ^1,2^, Silvia Battistoni ^1^, Daniela Montesarchio ^3^, Matteo Cocuzza ^1,4^, Simone Luigi Marasso ^1,4^, Alessio Verna,^4^, Laura Pasquardini ^5,6^, Roberto Verucchi ^7^, Lucrezia Aversa ^7^, Victor Erokhin ^1^, Pasquale D’Angelo ^1^, and Salvatore Iannotta ^1,*^

^1^IMEM – CNR Institute of Materials for Electronics and Magnetism, Parco Area delle Scienze 37/A, I-43124 ParmaItaly

^2^Physics Department and Ph.D. School on Material Science and Technology, University of Parma, Parma, Italy

^3^University of Naples ”Federico II” / Department of Chemical Sciences, 80126 Napoli, Italy

^4^Chilab - Materials and Microsystems Laboratory, DISAT, Politecnico di Torino, Chivasso (Turin), Italy

^5^Department of Industrial Engineering, Univ. of Trento, 38123 Trento, Italy

^6^Current address: Indivenire s.r.l., 38123 Trento, Italy.

^7^IMEM – CNR Institute of Materials for Electronics and Magnetism, Trento unit,c/o Fondazione Bruno Kessler, Viaalla Cascata 56/C, Povo, I-38123 Trento Italy

*salvatore.iannotta@imem.cnr.it

# Graphene gate electrodes Characterization

Before starting the functional characterizations of OECT, a morphological and structural characterization of the gate electrode has been performed. SEM characterization (**Fig. S 1**) was obtained with 15 keV acceleration voltage of the electron beam and 5.5 mm working distance.


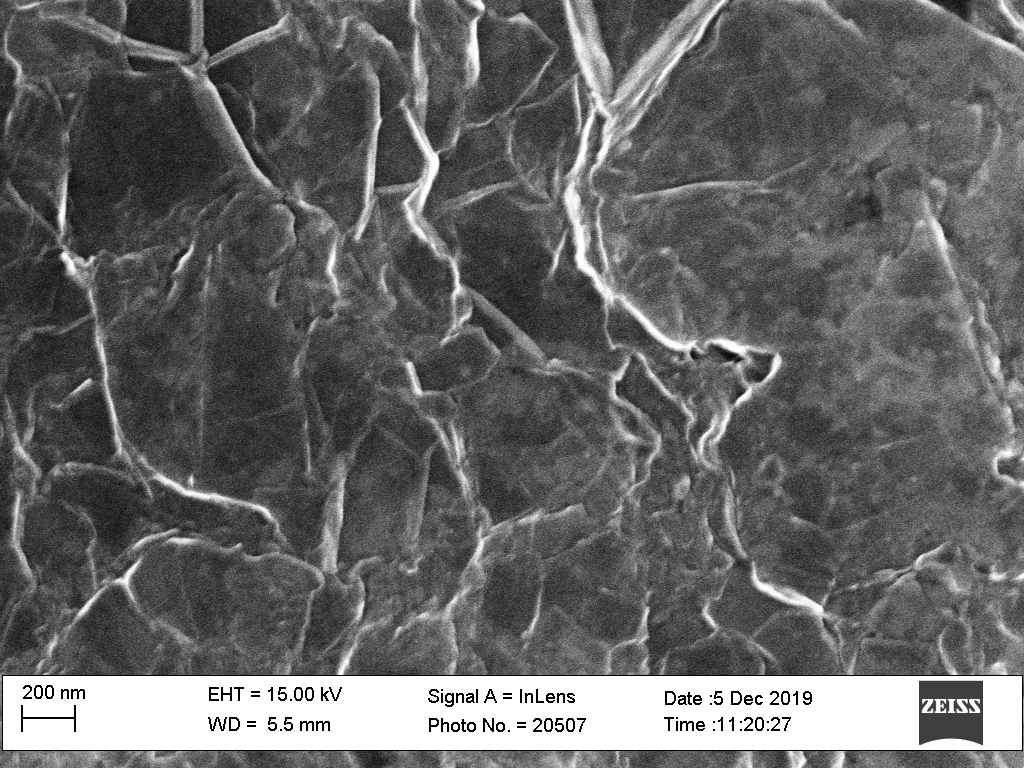


Fig. S 1: *SEM image of the graphene surface of the MLG-LDPE gate electrode*

The Raman analysis were collected with a 532 nm excitation laser, 50X objective, 0.5 mW of laser power, 10 s of acquisition time and two acquisitions per spectra. The Raman maps were acquired with similar parameters on an area of 20 µm x 18 µm with a spatial resolution of 500 nm.

Fig. S 2: *Raman spectra from different PMLG surface areas. Three typical main spectra were found and they are indicated with three different colors: green, red and black.*

Raman analysis reported in **Fig. S 2** shows the concurrent presence of visible peaks related to both MLG and LDPE. The latter were identified in the spectrum as PE Modes and they can be found at Raman shift values of: 1064 cm^-1^, 1140 cm^-1^, 1293 cm^-1^, 1412 cm^-1^, 2847 cm^-1^, 2886 cm^-1^ e 2933 cm^-1^. The peaks at 1064 cm^-1^ and 1140 cm^-1^ are related to the asymmetric stretching of C-C bonds of the polymer backbone. The peaks at 1293 cm^-1^ and 1412 cm^-1^ are associated to the asymmetric bending out-of-plane (twisting) and to the asymmetric bending in-plane (wagging) of the –CH_2_ group, respectively. Usually, Raman peaks in the region of the spectrum between 2700 cm^-1^ and 3100 cm^-1^ are identified as the signals due to the C-H bond stretching, thus peaks at 2833 cm^-1^ and 2933 cm^-1^ have been attributed to the bond asymmetric stretching, while the peak at 2847 cm^-1^ is connected to the asymmetric stretching of the C-H bond.

Graphene Raman peaks are located in the typical Raman shift values: 1347 cm^-1^, 1582 cm^-1^, 1610 cm^-1^ and 2700 cm^-1^ for a laser excitation at 532 nm. The peak at 1347 cm^-1^ is the so-called D-band, which can be found only in disordered graphene with a high density of defects, comes from the breathing mode A1g of the carbons in the benzene rings. The peak at 1582 cm^-1^ is the G-band, which is the only due to a normal first order Raman scattering process in graphene and is related to a primary in-plane vibrational mode (E2g) at the Brillouin zone center. D-band at 1610 cm^-1^ is another disorder-induced peak caused by double resonance intra-valley scattering of a photo-excited electron by a phonon along with elastic scattering by a defect. The peak at 2700 cm-^1^ is related to a second-order overtone of the D-band, but it does not need defects to be activated.

The Raman analysis is a powerful tool for distinguishing graphene from graphite structure. In our case, the 2D band is not a single peak but shows the presence of two different peaks, furthermore, it is less intense than the G-peak, indicating the presence of several graphene layers. The ratio between the intensity of the 2D and G peaks, I2D/IG, is about 0.55, which corresponds to a number of layers between 5 and 10.

From Raman analysis of different PMLG surface areas, we identified zones in which the ratio between the MLG G peak (1580 cm^-1^) and the LDPE C-H stretching mode intensities (2800 cm^-1^-2900 cm^-1^) significantly differs. The spectra obtained in these different areas are reported in **Fig. S 2** with different colors. According to the ratio values, it is possible to define three main different areas:

1- G Band >> LDPE modes (red line)

2- G-Band ≈ LDPE modes (black line)

3- G Band << LDPE modes (green line)

These results indicate a different depth thickness of the MLG in the LDPE matrix, in total agreement with the non-planar structure highlighted in the SEM analysis.

# Holes transport Characterization

In order to obtain a highly stable and fast responding OECT, it is necessary to characterize the temporal response of the drain current to any chemical or physical change into the device. The temporal response of an OECT depends on either both the ions transport from the electrolyte to the channel and the transit time of holes in the PEDOT:PSS channel. The latter can be estimated by calculating the time response of the OECT when driven by a constant gate current, I_gs_ [[1](#_ENREF_1)]:

$$\frac{dI_{ds}}{dt}= \frac{-I_{gs}}{\tau_{e}}$$

where τ_e_ is the holes transit time, which in the OECT engineered for this work has been found to be equal to 52.20 µs. **Fig. S 3** shows dI_ds_/dt calculated for different I_gs_ values (black hollow circles) and the corresponding linear fit (red dashed line), the slope of which corresponds to τ_e_. The value obtained is low enough to indicate that the holes transport in the channel is not the limiting factor in the OECT temporal response.


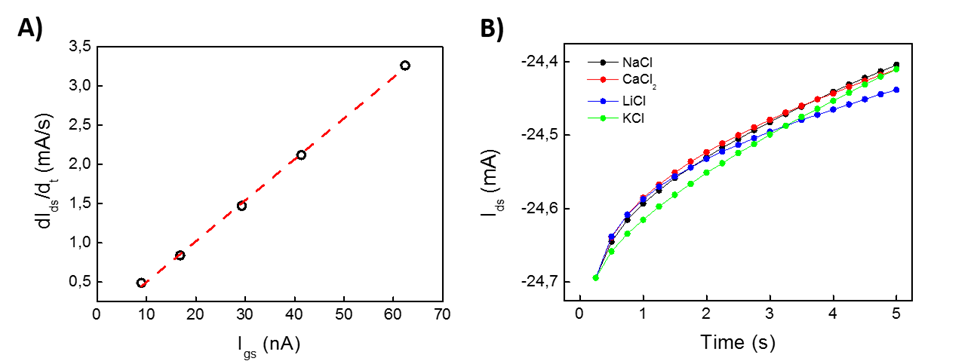


Fig. S 3: *Evaluation of the holes transit time τ_e_ which corresponds to the slope of the linear fit (red dashed line) calculated for different I_gs_ values (black circles).*

This means that the OECT temporal response is dominated by the ions transport from the electrolyte into the organic channel, according to the dependence of the drain current by the type of ion in the electrolyte solution.

# CHARACTERIZATION OF GOLD NANOPARTICLES


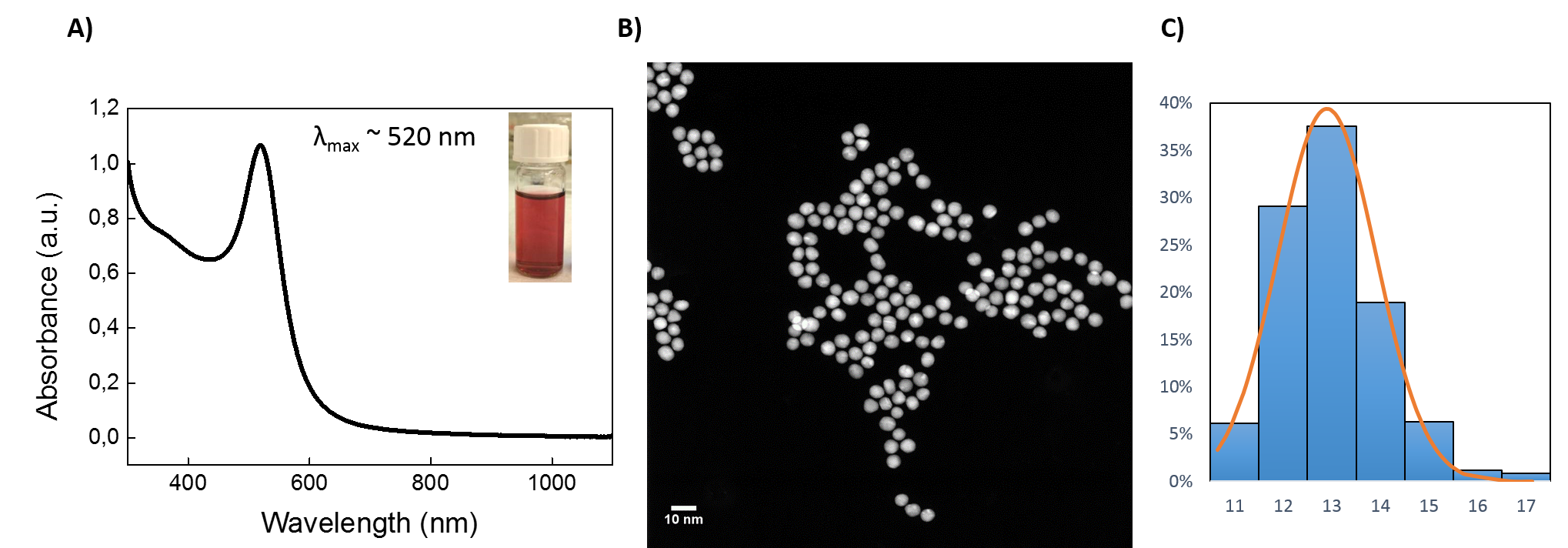


Fig. S 4: *a) Uv-vis spectrum of AuNPs dispersion synthesized trough seed-induced growth method; the inset shows the final aspect of the colloidal solution that appears as a clear red solution. b) typical TEM micrograph of a portion of AuNPs where the principal geometry is spherical c) size distribution of AuNPs having a mean diameter of about 13 nm.*


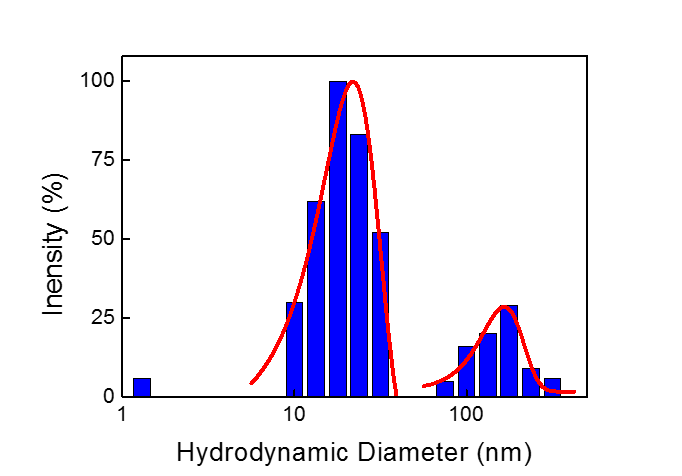


Fig. S 5: *Bimodal particle size distribution obtained for the analysis of the dynamic light scattering of the synthesized AuNPs in solution*

The production of AuNPs is confirmed by UV-vis absorbance measurement that shows the characteristic sharp absorbance peak around 520 nm, as reported in **Fig. S 4 a)**. The narrow peak and the absence of other peaks above 550 nm, both indicate the monodispersivety of the nanoparticles. This result is further confirmed by the TEM analysis; in fact, as it can be seen in **Fig. S 4 b)** the geometry of the AuNPs is mainly spherical and the size distribution calculated for a batch of 500 AuNPs shows a narrow dispersivity between 10 and 20 nm, with a mean diameter of about 13 nm (**Fig. S 4 c)** ).

Dynamic light scattering (DLS) has been performed to determinate the size of the AuNPs in solution and the results are reported in **Fig. S 5**. The hydrodynamic diameter as obtained by DLS shows a bimodal particles size distribution, which appears as two distinct peaks: a mode at small sizes (17-20 nm) related to a large number of particles and a second, less populated mode, at larger sizes (100-130 nm). The DLS first mode, with smaller size, gives a diameter that is larger than the size measured by TEM because DLS is based on the measure of the diffusion rate that depends not only on the core size of the nanoparticles but also on any surface structures including the capping agent. The second mode with higher hydrodynamic diameter is due to the presence of AuNPs aggregates.

A Zeta-potential ζ analysis has been performed in order to evaluate the nanoparticles charge and stability. The zeta potential cannot be measured directly, but is estimated using the Hückel equation:

$$\mu= \frac{2\varepsilon f(\kappa a)}{3\eta}$$

where µ is the electrophoretic mobility, ε is the dielectric constant, η the viscosity of the surrounding liquid and f(κa) is the Henry’s function in which κ is the Debye length and *a* is the particle radius. The resulting effective surface potential of the citrate-capped AuNPs is -13.44 mV.

# DECORATION OF A PMLG ELECTRODE WITH AuNPs

Since the citrate-capped AuNPs in solution possess a net negative charge, we exploited the electrophoretic deposition method (EPD) to deposit the AuNPs on the surface of the PMLG electrode. In the EPD technique, charged nanoparticles are driven towards and deposited onto the surface of an electrode, dip-coated in a solution, with the application of an external electric field perpendicular to the substrate. To deposit the AuNPs, a positive potential of 40 V was applied constantly at the PMLG acting as anode electrode while a grounded chrome electrode was used as cathode.


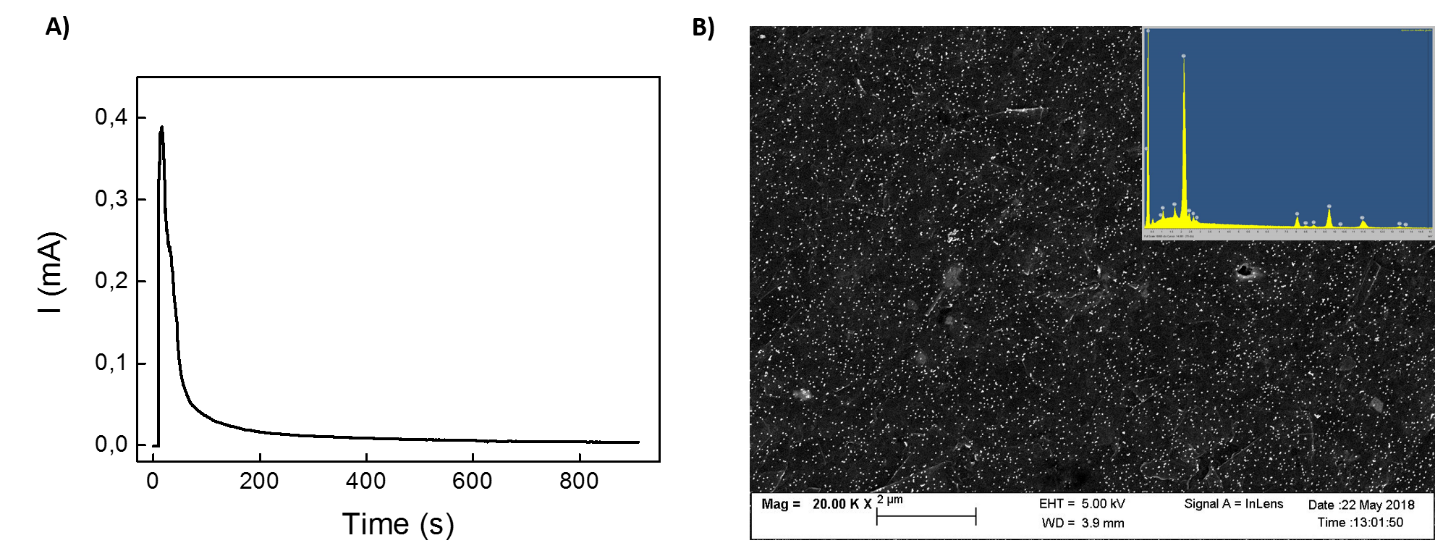


Fig. S 6: *a) The electric field between the anode and the cathode drives AuNPs towards the PMLG surface, where they are deposited producing a specific I vs time profile. b) SEM image of the PMLG gate electrode surface after an homogenous deposition of AuNPs; in the inset the EDX analysis of the as decorated gate electrode surface*

The current measured in an EPD experiment (**Fig. S 6 a)**) is indicative of the motion and deposition of the charge-carrying nanoparticles. In fact, when the positive potential is switched on an intense current is recorded, the value of which depends on the AuNPs initial concentration. Subsequently, the current gradually drops down with the decreasing of the density of AuNPs in solution due to their deposition on the PMLG electrode until it reaches zero indicating the end of the reaction.

To investigate the morphology of the PMLG electrode surface after the AuNPs deposition, a SEM micrograph was taken (**Fig. S 6 b)**. A well-dispersed and homogenous distribution of AuNPs on the PMLG surface is observed, where the nanoparticles retain the shape and the size of the solution, proving that the EPD method does not affect their physical properties. The estimated coverage percentage of the PMLG surface is 42%.

A study of the elemental composition of the AuNPs-decorated PMLG electrode surface was carried out with the Energy Dispersive X-ray analysis (EDX) to further confirm the presence AuNPs. In the inset of **Fig. S 6 b)** the spectrum shown clearly presents the main gold signals at 1.87 KeV, 2.12 KeV (M Line) and 9.89 KeV (L Line), in addition to the carbon signal 0.28 KeV (K line) related to the graphene substrate, thus confirming the presence of AuNPs on the gate electrode surface. The peaks at 0.930 and 8.040 KeV are due to the copper emission, which is the constituent material of the sample holder.

# Aptamers


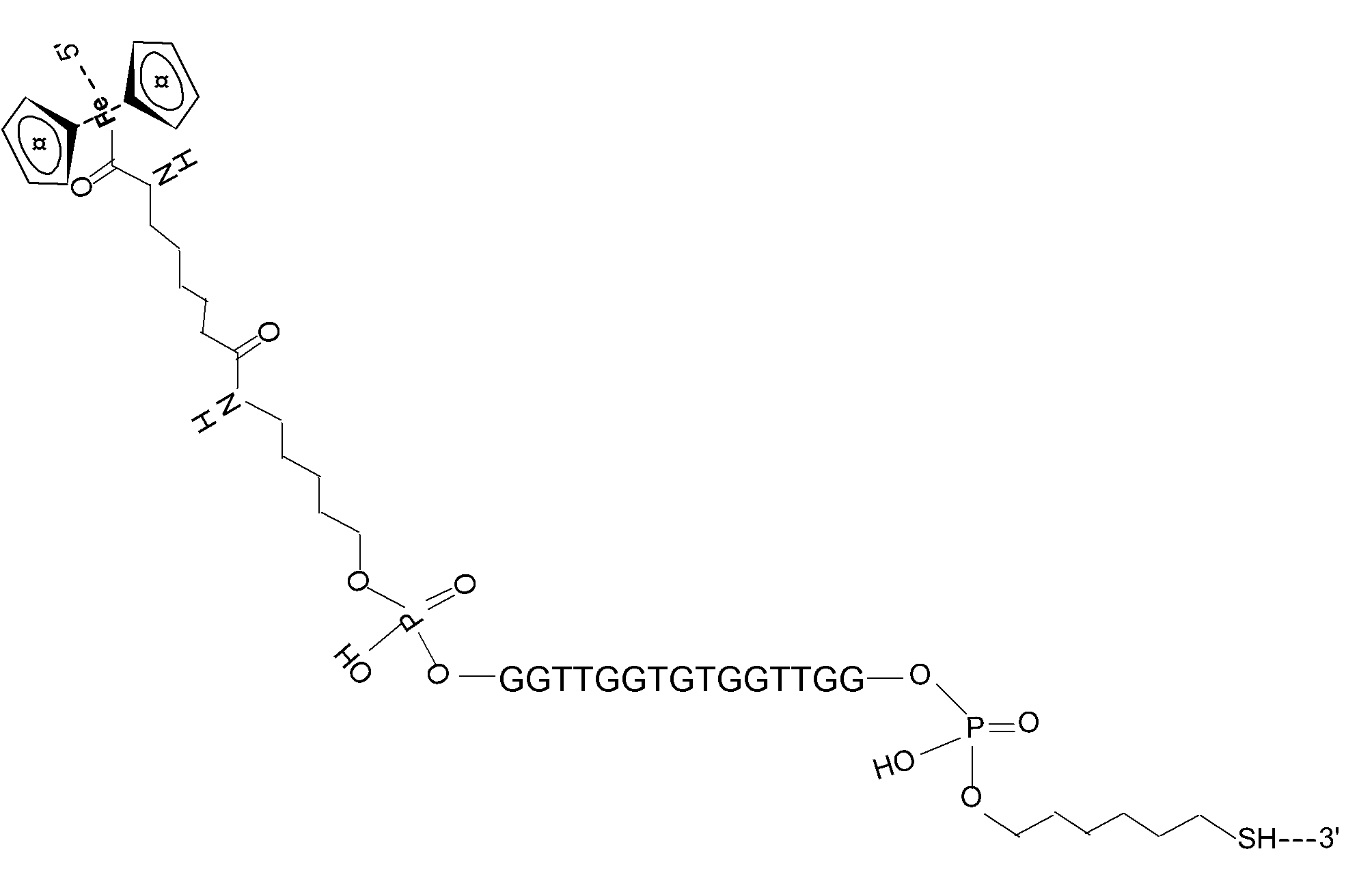


Fig. S 7: Modifications inserted at the 5’ (Fc, on the top) and 3’ (C6-SH, on the bottom) end of the TBA15 oligonucleotide 5’ Fc-GGTTGGTGTGGTTGG-C6-SH 3’, synthesized by BIOMERS (Germany) ^[[1]](#footnote-1)^. Image created with ChemSketch (https://www.acdlabs.com/products/draw_nom/draw/chemsketch/)

# FUNCTIONALIZATION OF AuNPs-PMLG GATE ELECTRODE BY IMMOBILIZATION OF THROMBIN BINDING APTAMER-15 (TBA-15)

X-ray photoelectron spectroscopy (XPS) analysis has been also performed to study in more details the formation of the gold-thiol binding between aptamers and AuNPs on the PMLG gate electrode surface.

The XPS studies have been performed on three samples: the bare PMLG electrode, the AuNPs-PMLG electrode and the AuNPs-PMLG that has been functionalized with an aptamer solution having the chosen oligonucleotides concentration of 1 µM prepared with the same approach used for the gate electrode preparation.

Table 1: *atomic percentages for all elements in the three samples, as evaluated by XPS analysis.*

|  | C graphene | Au | C Aptamer | O | N | P | S |
| --- | --- | --- | --- | --- | --- | --- | --- |
| *PMLG* | 95.5 | - | - | 4.5 | - | - | - |
| *PMLG-AuNPs* | 95.3 | 0.04 | - | 4.4 | - | - | - |
| *PMLG-AuNPs-TBA-15* | 19.8 | 0.08 | 64.6 | 11.4 | 2.7 | 1.1 | 0.4 |

The atomic percentages of all elements present on the three gate electrodes were evaluated and the relative data are shown in **Table 1**. In the first two samples, the main element is carbon (C1s core level), as expected due to the presence of graphene as substrate, while, in the aptamers functionalized electrode, it is possible to distinguish carbon core C1s core level signals coming from the graphene and organics compounds. In this case, the attenuation of the graphene signal suggests that the mean thickness of the organic over-layer could be around 6nm in case of a complete and homogeneous coverage of the graphene substrate by the aptamers layer[[2](#_ENREF_2)]. No further consideration concerning organic film thickness based on Au4f signal attenuation can be done, due to the very low metal S/N ratio. Presence of aptamers fingerprints confirms the achieved functionalization process, while it is difficult to discuss the absolute C, O, N, P and S atomic percentages due to the low signal to noise ratio of the last 3 elements. Indeed, this is a common issue as suggested by the large variability reported in literature for aptamers[[3-5](#_ENREF_3)].

By studying in details the different core level lineshapes, information regarding the chemical/physical properties of the three surfaces are achieved. T**able 2** summarizes the results for all core levels analysis, where single components binding energy and description are shown.

Table 2: *Core level components for C1s, O1s, Au4f, N1s, P2p, S2p in all samples. Each component is identified by its chemical origin and binding energy, BE, in eV.*

|  |  | Peak binding energy, BE (eV) | | |
| --- | --- | --- | --- | --- |
| Core level | **Chemical species** | *PMLG* | *PMLG-AuNPs* | *PMLG-AuNPS-TBA-15* |
| *C1s* |  |  |  |  |
|  | *Graph. C-C* | 288.44 | 287.75 | 284.62 |
|  | *Graph. C-O* | 289.31 | 288.65 | 285.52 |
|  | *Apt. C-C, C-H* |  |  | 285.21 |
|  | *Apt. C-N, C-O* |  |  | 286.16 |
|  | *Apt. N-C=O* |  |  | 287.00 |
|  | *Apt. C-NH2* |  |  | 287.79 |
|  | *Apt. satellite* |  |  | 288.71 |
| *O1s* |  |  |  |  |
|  | *Graph. C-O* | 534.93 | 534.51 |  |
|  | *Apt. C=O, N-C=O, P-O, C-O* |  |  | 532.04 |
|  | *Apt. C-O-C, C-OH* |  |  | 533.54 |
| *Au 4f* |  |  |  |  |
|  | *Au 4f 7/2* |  | 86.70 | 84.00 |
|  | *Au 4f 5/2* |  | 90.40 | 87.66 |
| *N1s* |  |  |  |  |
|  | *Apt. N-C* |  |  | 399.6 |
|  | *Apt. N-H, N-H2* |  |  | 400.82 |
|  | *Apt. N-(C)_3_* |  |  | 401.82 |
| *P2p 3/2* |  |  |  |  |
|  | *Apt. P-O* |  |  | 134.29 |
| *S2p 3/2* |  |  |  |  |
|  | *Apt. S-Au* |  |  | 161.80 |

In the PMLG sample, C1s main peak is broad (1.3eV) and located at 288.44 eV ( **Fig. S 8 a)**), about +4 eV from the expected ~284.7eV value for sp^2^-hydbridized graphitic carbon. As previously stated, this is due to the insulating character of the PMLG substrate that leads to formation of surface charges during XPS analysis. A second peak at 289.31eV is also present and it is related to C-O species on graphene[[6](#_ENREF_6), [7](#_ENREF_7)]. The corresponding oxygen species for O1s emission is located at 534.93eV (**Fig. S 8 b)**). The absence of any C1s peak asymmetry, typical of a single or of a few layer graphene[[6-8](#_ENREF_6)], confirms the multilayered nature of the electrode, also taking into account the broadening due to charging.


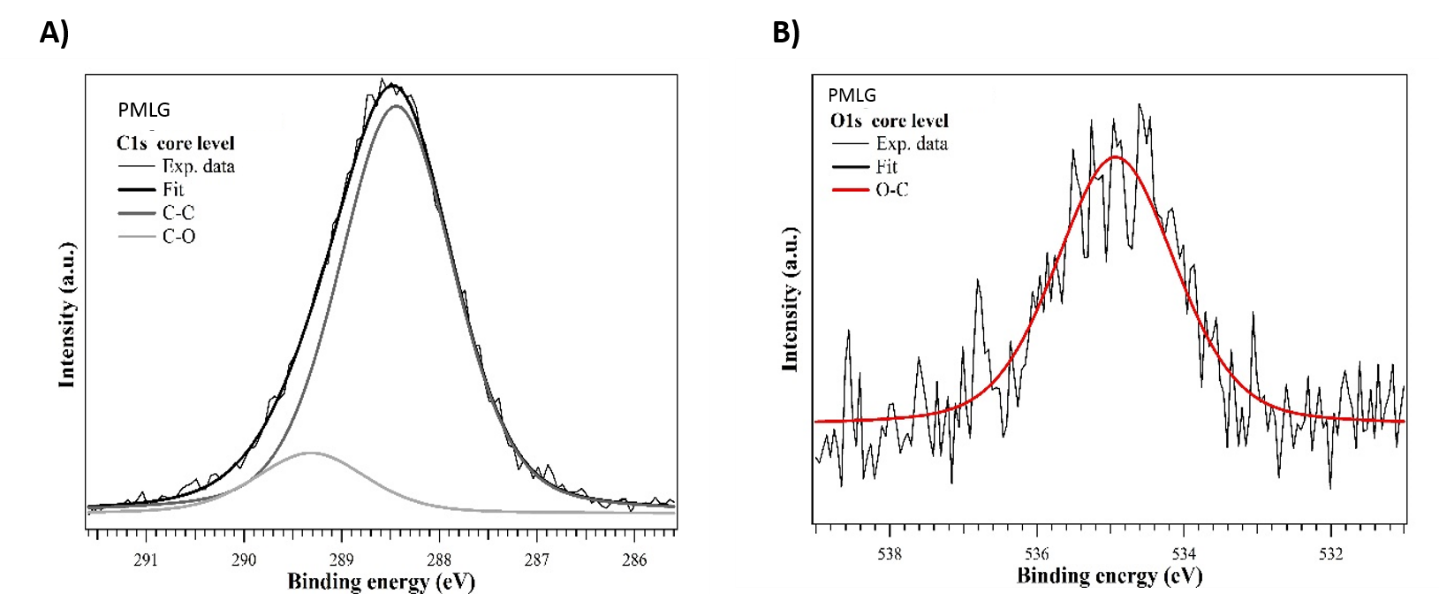


Fig. S 8: C1s and O1s core levels (left, right) for Graphene on PMLG sample. Single components are described in the legends.

Similar results have been found for PMLG-AuNPs electrode, with a slightly lower BE shift of about +3.5eV. C1s and O1s show same features of pure graphene (**Fig. S 9 a) and b)**), while Au4f is characterized by a doublet (**Fig. S 9 c)**), i.e. two peaks separated by 3.6eV as expected for gold. The very low signal to noise ratio is related to the low atomic percentage for gold, about 0.04% that is close to the XPS detection limit.


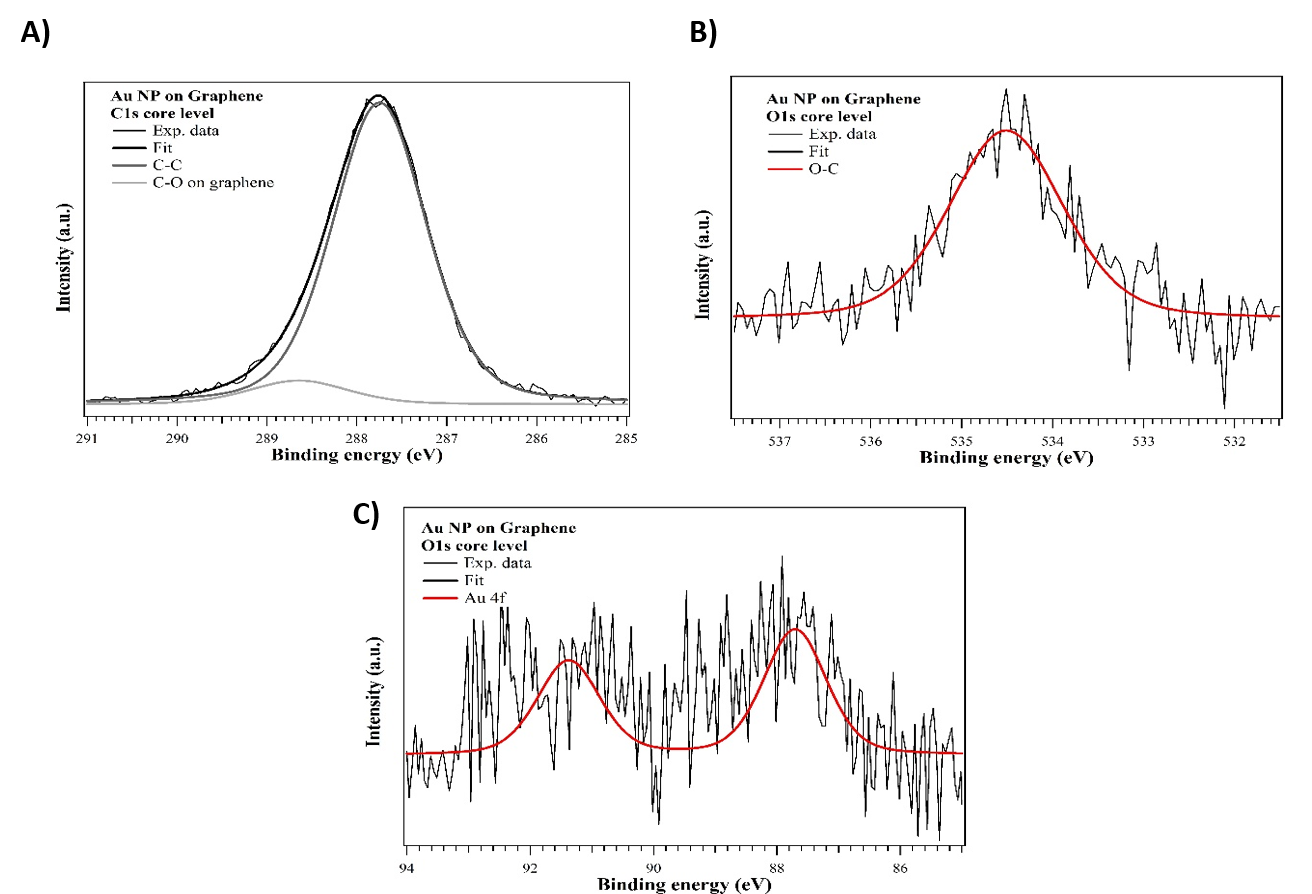


Fig. S 9: a) C1s, b) O1s and c) Au4f core levels for PMLG-AuNPs electrode. Single components are described in the legends.

Results for PMLG-AuNPs-TBA-15 gate electrode are very different. As stated before, fingerprints of all expected elements in the aptamer composed of a chain of phosphates (P) and sugar with a sequence of 9 guanine and 6 thymine basis (C, O, N), linked with a thiol group (S), are present. As it can be seen upon comparison with previous results, lineshape of C1s and O1s peaks is quite different, suggesting presence of new chemical species (**Fig. S 10**).


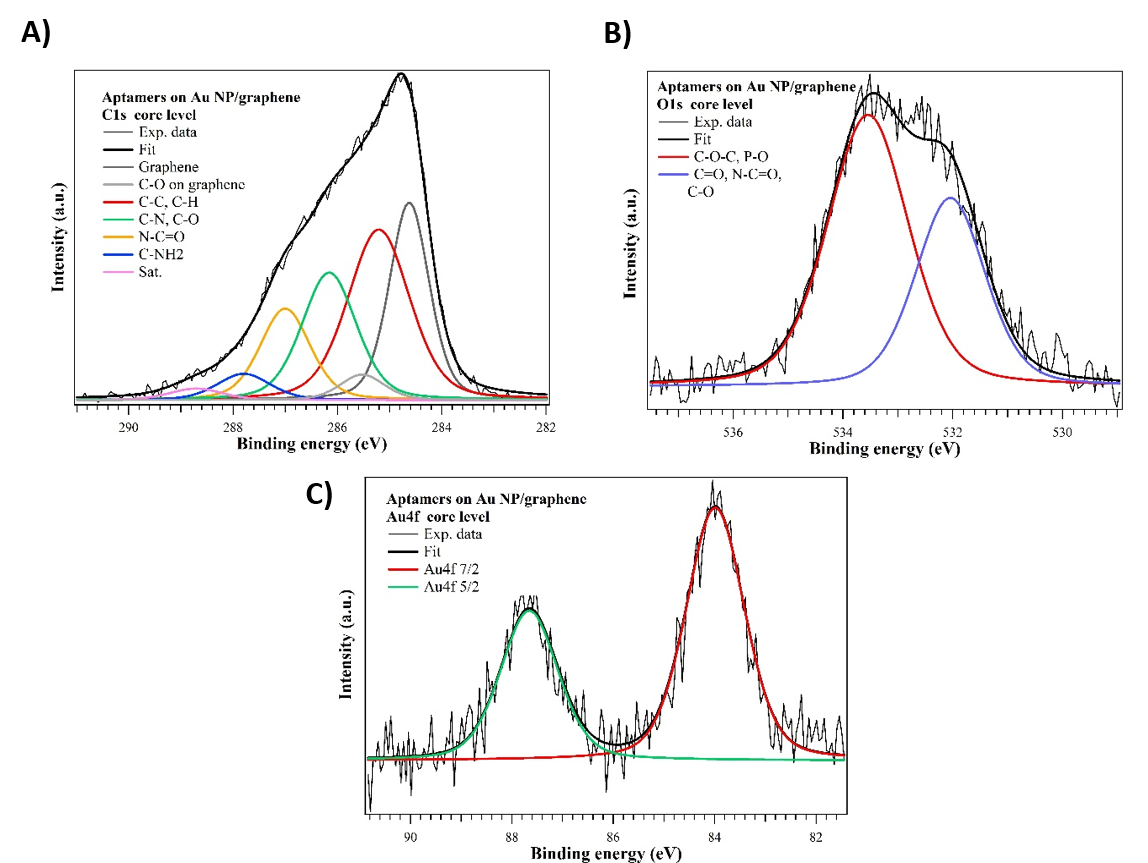


Fig. S 10: a) C1s, b) O1s and c) Au4f core levels for PMLG-AuNPs-TBA-15. Single components are described in the legends.

C1s peak has a broad (4eV) and structured line shape (**Fig. S 10 a)**). A signal coming from the graphene layer is still detectable at 284.62eV, at the expected BE value in absence of charging effects, as well as a small component from C-O species on graphene at 285.52eV. The peak full width at half maximum (FWHM) is about 0.9eV for both features, which is significantly lower than that found for virgin graphene on LDPE sample. A large (1.3eV) and intense structure is located at 285.21eV, it can be attributed to C-C and C-H species present in the alkyl chain, sugar groups, thymine and guanine basis. Their different chemical properties lead in C1s to the most intense component, as expected from the large number of these carbon atoms, but broad, due to the impossibility to discriminate the small BE differences of C-C and C-H in all groups[[9](#_ENREF_9)]. A second feature is present at 286.16eV (FWHM 1.23eV), located at about +1eV from the main C-C/C-H peak and thus related to both C-N species in the two basis[[10](#_ENREF_10)] and to C-O in sugar[[11](#_ENREF_11)]. Other two peaks, with decreasing intensity, are located at 287.00 (FWHM 1.13eV) and 287.79eV (FWHM 1.2eV); they can be attributed to carbon in N-C=O bond in guanine and thymine[[12](#_ENREF_12)] and in C-NH_2_ bond in guanine[[5](#_ENREF_5)]. A last peak is located at 288.71eV (FWHM 1.2eV) and it could be due to a shakeup electron promotion process typical of large molecules. Though characterized by a complex lineshape, the deconvolution of C1s peak shows different components with FWHM well above the system resolution (0.86eV) that corresponds to chemical species present in the studied system, i.e. aptamer on PMLG-AuNPs electrode, and are in good agreement with features found for O1s and N1s.

O1s peak analysis (**Fig. S 10 b)**) shows to broad (FWHM ~1.6eV) components located at 532.04 and 533.54 eV. Unlike the C1s peak, the deconvolution of O1s peak is always more difficult and it is not comprehensively discussed in literature[[13](#_ENREF_13), [14](#_ENREF_14)]. However, the two peaks are due to the superposition of the different oxygen chemical species, showing similar BE. In particular, the component at 532.04 eV can be due to oxygen in C=O and N-C=O in guanine and thymine, while a very small contribution from C-O on graphene cannot be excluded. The peak at higher BE can be identified as oxygen in C-O-C bond and in phosphates group[[15](#_ENREF_15)].

Concerning the main gold XPS signal, the higher concentration with respect to the previous sample leads to a better signal to noise ratio, with the two doublet peaks well defined at the expected BE of 84 eV for Au4f 7/2 ( **Fig. S 10 c)**). In fact, the functionalized surface does not show any charging effect during XPS analysis, probably due to the slightly higher gold content improving electrical conductivity with respect to previous sample.

Aptamers functionalization process should occur by interaction of gold with the thiol group. The S2p 3/2 peak (S2p is a doublet with a spin orbit splitting of 1.16 eV) is located at 161.80 eV and very broad (1.8 eV), as shown in **Fig. S 11 a)**. Although the presence of a second doublet around 162.5 eV cannot be excluded due to the low signal to noise ratio, the measured BE is typical of a surface bound Au-thiolate species[[16](#_ENREF_16)], while unbound thiols should be around 163 eV. This means that all or, at least, most of aptamers are covalently bound to Au NPs, which is a further evidence of an achieved functionalization process.


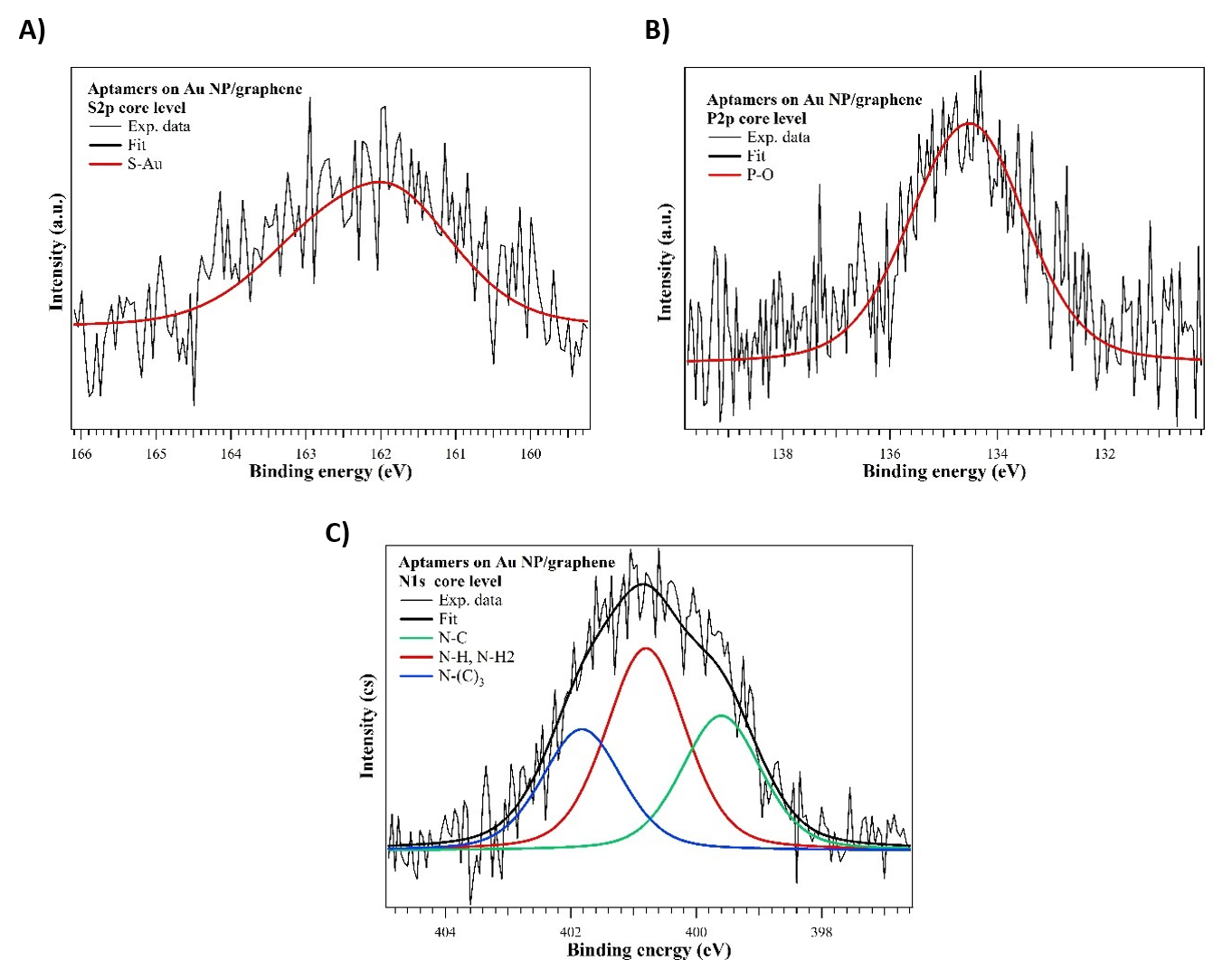


Fig. S 11: a) S2p, b) P2p and c) N1s core levels for PMLG-AuNPs-TBA-15. Single components are described in the legends.

P2p core level (**Fig. S 11 b)**) is characterized by a single peak, i.e. a doublet with spin orbit splitting of 0.84eV, located at 134.29eV and related to phosphates groups[[15](#_ENREF_15), [17](#_ENREF_17)].

N1s core level shows the presence of three main features (**Fig. S 11 c)**), all being representative of chemical species in guanine and thymine. A first one is located at 399.6 eV and is typical for nitrogen in aromatic rings, the most intense peak at 400.82 is representative of N-H and N-H_2_ groups, the most frequent type of nitrogen atom. The third one at 401.82eV can be attributed to nitrogen atom in graphitic environment, i.e. atoms linking each basis with the sugar group[[18](#_ENREF_18), [19](#_ENREF_19)].

# Final Image of the aptasensor


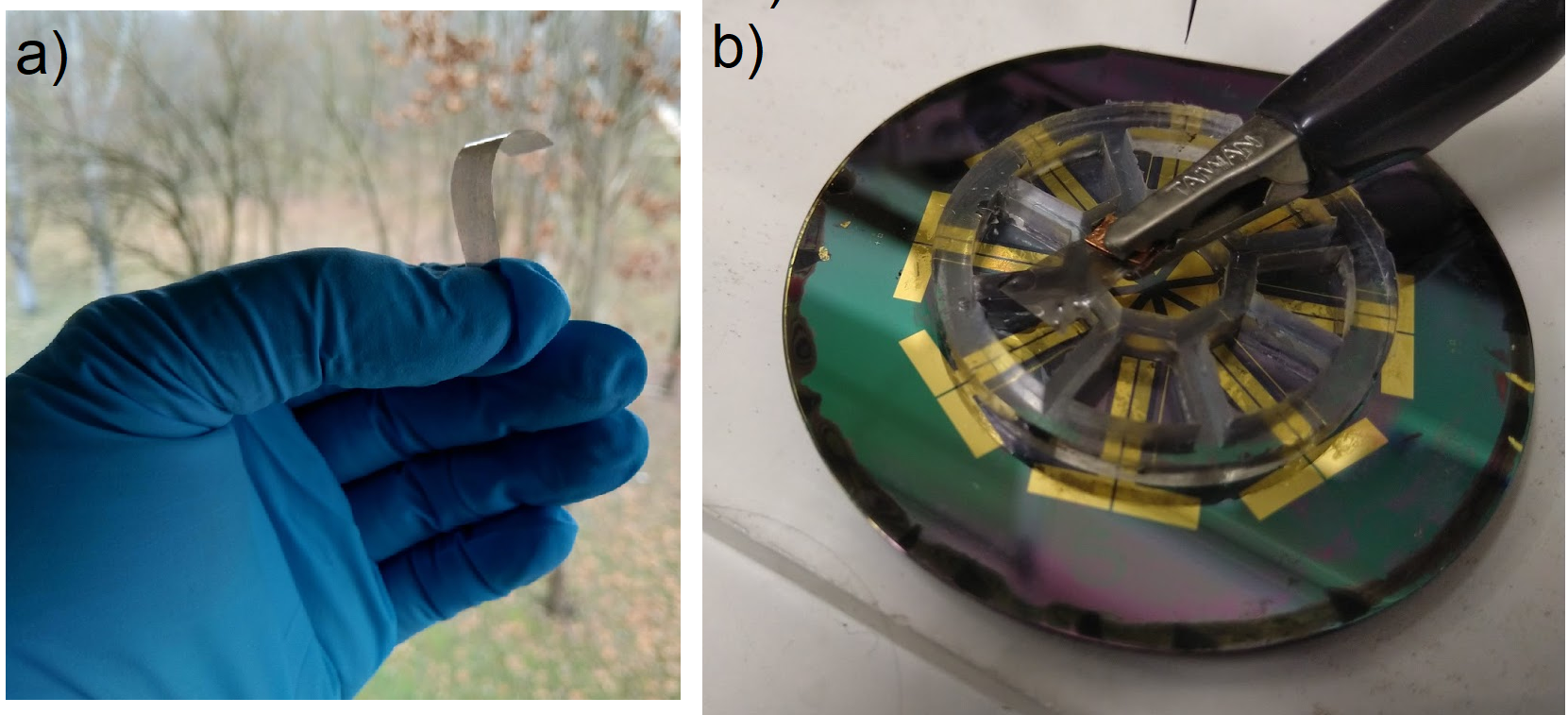


Fig. S 12: panel a) PMLG electrode and panel b) PMLG gated OECT.

# The reproducibility of OECTs





**Fig. S13: Normalized transfer curves (left) for two devices sharing the same channel aspect ratio; percentage current difference (right) between the same devices**

Reproducibility tests have been performed by recording the OECT response for homologue devices in terms of channel aspect ratio. Normalized transfer curves (Fig. S13, left panel) are almost indistinguishable over the whole gate voltage range. The difference between the channel currents of the analyzed devices, expressed in percentage, is also shown (Fig. S13, right panel); the maximum deviation (at V_gs_=-0.2V) is in the order of a few percent and tends to decrease as the de-doping of the PEDOT:PSS channel becomes even more effective.

1. Khodagholy, D., et al., *High transconductance organic electrochemical transistors.* Nature communications, 2013. **4**: p. 2133.

2. Hüfner, S., *Photoelectron spectroscopy: principles and applications*. 2013: Springer Science & Business Media.

3. Furukawa, M., et al., *Geometrical characterization of adenine and guanine on Cu (1 1 0) by NEXAFS, XPS, and DFT calculation.* Surface Science, 2007. **601**(23): p. 5433-5440.

4. Wang, X., et al., *Effective surface functionalization of nanocrystalline diamond films by direct carboxylation for PDGF detection via aptasensor.* ACS applied materials & interfaces, 2012. **4**(7): p. 3526-3534.

5. Zhang, F., et al., *Graphene covalently modified by DNA G-Base.* The Journal of Physical Chemistry C, 2013. **117**(7): p. 3513-3519.

6. Longo, A., et al., *Graphene oxide prepared by graphene nanoplatelets and reduced by laser treatment.* Nanotechnology, 2017. **28**(22): p. 224002.

7. Pontiroli, D., et al., *Tracking the hydrogen motion in defective graphene.* The Journal of Physical Chemistry C, 2014. **118**(13): p. 7110-7116.

8. Tatti, R., et al., *Synthesis of single layer graphene on Cu (111) by C 60 supersonic molecular beam epitaxy.* RSC Advances, 2016. **6**(44): p. 37982-37993.

9. Liu, L., et al., *Electrochemistry of a C84-C 2 (IV)-Modified Electrode in Aqueous Solutions and Its Interaction with Guanine.* The Journal of Physical Chemistry C, 2011. **115**(13): p. 5966-5973.

10. Nardi, M., et al., *Electronic properties of tetrakis (pentafluorophenyl) porphyrin.* New Journal of Chemistry, 2013. **37**(4): p. 1036-1045.

11. Jia, Y. and F. Li, *Studies of Functional Nucleic Acids Modified Light Addressable Potentiometric Sensors: X-ray Photoelectron Spectroscopy, Biochemical Assay, and Simulation.* Analytical chemistry, 2018. **90**(8): p. 5153-5161.

12. Chung, S., et al., *Magnetic force assisted electrochemical sensor for the detection of thrombin with aptamer-antibody sandwich formation.* Biosensors and Bioelectronics, 2018. **117**: p. 480-486.

13. Li, T., et al., *Gold nanoparticles/Orange II functionalized graphene nanohybrid based electrochemical aptasensor for label-free determination of insulin.* Rsc Advances, 2016. **6**(36): p. 30732-30738.

14. Furukawa, M., et al., *Geometrical characterization of pyrimidine base molecules adsorbed on Cu (110) surfaces: XPS and NEXAFS studies.* Surface science, 2003. **532**: p. 261-266.

15. Khattak, G., A. Mekki, and L. Wenger, *X-ray photoelectron spectroscopy (XPS) and magnetic susceptibility studies of vanadium phosphate glasses.* Journal of Non-Crystalline Solids, 2009. **355**(43-44): p. 2148-2155.

16. Castner, D.G., K. Hinds, and D.W. Grainger, *X-ray photoelectron spectroscopy sulfur 2p study of organic thiol and disulfide binding interactions with gold surfaces.* Langmuir, 1996. **12**(21): p. 5083-5086.

17. Deng, J., et al., *Heparin/DNA aptamer co-assembled multifunctional catecholamine coating for EPC capture and improved hemocompatibility of vascular devices.* Materials Science and Engineering: C, 2017. **79**: p. 305-314.

18. Qu, D., et al., *Formation mechanism and optimization of highly luminescent N-doped graphene quantum dots.* Scientific reports, 2014. **4**: p. 5294.

19. Liu, Y., et al., *A label-free photoelectrochemical aptasensor based on nitrogen-doped graphene quantum dots for chloramphenicol determination.* Biosensors and Bioelectronics, 2015. **74**: p. 1016-1021.

1. <https://www.biomers.net/de/Katalog/Modifikationen> [↑](#footnote-ref-1)
